# Stress‐induced phosphoprotein 1 restrains spinal cord ischaemia‐reperfusion injury by modulating NF‐κB signalling

**DOI:** 10.1111/jcmm.17030

**Published:** 2021-11-03

**Authors:** Hongdou Jin, Xin Ge, Zhirong Huan, Hao Yao, Ce Xu, Jimin Cai

**Affiliations:** ^1^ Department of General Surgery Wuxi 9th Hospital Affiliated to Soochow University Wuxi City Jiangsu Province China; ^2^ Department of ICU Wuxi 9th Hospital Affiliated to Soochow University Wuxi City Jiangsu Province China

**Keywords:** ischaemia‐reperfusion, microglia, NF‐κB signalling, rat, spinal cord injury, stress‐induced phosphoprotein 1

## Abstract

Spinal cord injury (SCI), a major cause of disability, causes high global disease and economic burdens. Stress‐induced phosphoprotein 1 (STIP1) has been identified to be involved in spinal cord ischaemia‐reperfusion injury (SCII); however, the effect of STIP1 on SCII remains unclear until now. This study aimed to examine the role of STIP1 in SCII and unravel the possible mechanisms. Western blotting and immunohistochemical staining showed that STIP1 expression rapidly increased and then decreased in rat spinal cord following SCII treatment. Neurological function scoring, HE staining, immunohistochemical staining and Western blotting revealed that STIP1 overexpression alleviated SCII‐induced motor dysfunction of hind limbs, neuronal loss and inflammation in spinal cord, and inhibited activity of nuclear factor kappa B (NF‐κB) signalling in rats. Immunoprecipitation identified that STIP1 was co‐located with Iba‐1. In addition, STIP1 was found to ameliorate oxygen and glucose deprivation (OGD)‐induced inflammation and activation of NF‐κB signalling in mouse microglia BV2 cells, and STIP1 resulted in decrease of heat shock protein family A member 8 (HSPA8), increase of IκBβ expression and reduced binding of IκBβ to HSPA8 in BV2 cells. The results of the present study demonstrate that STIP1 alleviates ischaemia/reperfusion‐induced neuronal injury and inflammation in rat spinal cord and mouse microglial cells by deactivating NF‐κB signalling. These findings may provide novel insights for the clinical diagnosis and treatment of SCI.

## 1 INTRODUCTION

Spinal cord injury (SCI), a devastating neurological state, is a major cause of disability.^1^ For the last 30 years, the global prevalence of SCI has increased from 236 to 1298 cases per million populations.^2^ Results from the Global Burden of Diseases, Injuries and Risk Factors (GBD) Study 2016 showed that there were 27.04 million (95% uncertainty interval, 24.98–30.15 million) patients living with SCI and 0.93 million (95% uncertainty interval, 0.78–1.16 million) new cases diagnosed with SCI across the world in 2016, and this disorder was estimated to cause 9.5 million (95% uncertainty interval, 6.7–12.4 million) years of life lived with disability in 2016.^3^ More importantly, the global number of prevalent cases with SCI is estimated to rise because of the population growth.^4^ As a potential risk of developing debilitating and even life‐threatening secondary conditions, SCI poses high disease, social and economic burdens throughout the world.^5^

As a traumatic event, SCI may cause motor, sensory and autonomic dysfunctions,^6^ and its pathophysiology comprises acute and chronic phases, and incorporates a cascade of destructive events such as ischaemia, oxidative stress, inflammatory events, apoptotic pathways and locomotor dysfunctions.^6^ The most vulnerable clinical manifestation after injury is the interruption of spinal cord vascular supply and hypotension, leading to spinal cord ischaemia.^7^, ^8^ Ischaemia‐reperfusion is reported to induce reactive oxygen species production, mitochondrial dysfunction, ion homeostasis imbalance, inflammatory and neural apoptosis,^9^ and inflammation, oxidative stress response and cytotoxicity could be mediated by microglial cells.^10^ It has been reported that microglia is activated in mouse spinal cord following ischaemia‐reperfusion treatment, and minocycline, a macrolide antibiotic, was found to inhibit microglial activation, preserve hind limb motor function and restrain inflammatory factor production in mice with spinal cord ischaemia‐reperfusion injury (SCII).^11^ Microglia is therefore hypothesized as a therapeutic target for SCII.

Stress‐induced phosphoprotein 1 (STIP1), is an adaptor protein that assists the transfer of protein from heat shock protein 70 (HSP70) to HSP90 by binding both HSP90 and substrate‐bound HSP70,^12^, ^13^ has been found to bind to other chaperones and modulate their activities in addition to HSP70 and HSP90, and aberrant STIP1 expression has been shown to lead to unfolding protein responses.^14^, ^15^, ^16^ STIP1 was reported to bind to prion protein to inhibit binding of soluble amyloid‐β oligomers to prion protein or mouse primary hippocampal neurons, and STIP1 treatment prevents amyloid‐β‐induced synaptic loss and neuronal death in mouse cultured neurons and long‐term potentiation inhibition in mouse hippocampal specimens.^17^ In addition, elevated STIP1 expression was detected in cerebral specimens of humans and rats with ischaemic stroke, and STIP1 overexpression promoted recruitment of bone marrow–derived cells to ischaemic brain, and facilitated neurological recovery.^18^ In heterozygous STIP1 knockout mice, aggravated ischaemic damage was seen in brain, and extracellular STIP1 treatment prevented ischaemia‐mediated neuronal cell death, indicating the neuronal protective role of STIP1.^19^ Recently, STIP1 expression was found to be increased firstly followed by a decrease in rabbit spinal cord specimens after SCII,^20^ suggesting that STIP may be involved in SCII. However, the effect of STIP1 on SCII remains unclear until now. This study was therefore designed with aims to examine the role of STIP1 in SCII and unravel the possible mechanisms.

## 2 MATERIALS AND METHODS

### 2.1 Cell culture and treatment

Mouse microglia BV2 cells were purchased from Procell (Wuhan, China), and cultured with minimum essential medium (Sigma‐Aldrich; St. Louis, MO, USA) supplemented with 10% foetal bovine serum (Sigma‐Aldrich, St. Louis, MO, USA) in a homothermal incubator containing 5% CO_2_. For oxygen and glucose deprivation (OGD) treatment, cells were incubated in glucose‐free medium (Sigma‐Aldrich, St. Louis, MO, USA) with 95% N_2_ and 5% CO_2_ for 6 h, and recovered for 24 h.

### 2.2 Animal modelling and grouping

Healthy 8‐week‐old male rats of the SD strain were purchased from the Laboratory Animal Center of Yangzhou University (Yangzhou, China) and housed in a SPF‐grade facility under controlled conditions (12 h/12 h light/dark cycles, 22 ± 1°C) with free access to food and water. After acclimatization for 1 week, rats underwent SCII surgery as described previously.^21^ Prior to surgery, rats were randomly assigned into four groups, of 6 animals in each group. Rats in the SCII group were given SCII surgery, animals in the SCII + LV group were given empty lentivirus (Hunan Fenghui Biotechnology Co., Ltd., Changsha, China) by intrathecal injection followed by SCII surgery, and rats in the SCII + LV‐STIP1 group were intrathecally injected with the pLVX‐IRES‐puro vector (Hunan Fenghui Biotechnology Co., Ltd., Changsha, China) containing the coding sequence of STIP1 (1 × 10^7^ TU; File S1), followed by SCII surgery, while rats in the Sham group received abdominal aorta separation without clip closure alone. Rats received surgery 3 days post‐injection with lentivirus. Following induction of anaesthesia by intraperitoneal injection of 50 mg/kg sodium pentobarbital and abdominal shaving, rats received laparotomy. Briefly, the rat abdominal aorta was exposed, and heparin (130 U/kg) was intravenously given to prevent blood clotting. Clamping was performed for 1 h and reperfusion for 0, 12 or 24 h. The rat body temperature was maintained at 36.5 ± 0.5°C. After clamping for 1 h and reperfusion for 24 h, the rats received neurological function scoring and sacrifice, and spinal cord (L2–L5) specimens were sampled for subsequent detections.

### 2.3 Neurological function scoring

Following induction of ischaemia‐reperfusion for 24 h, the motor function of rat hind limbs was recorded in each group according to the Basso, Beattie and Bresnahan (BBB) score.^21^, ^22^ According to the presence of motor function defect, scores were assigned ranging from 0 to 21 points. Scores were evaluated by two investigators who were blinded to the grouping.

### 2.4 Western blotting

Total protein was extracted from spinal cord specimen with RIPA lysis buffer (Beyotime, Haimen, China) with 1% phenylmethanesulfonyl fluoride (PMSF; Beyotime), and nuclear and cytoplasmic proteins were extracted with Nuclear and Cytoplasmic Protein Extraction Kit (Beyotime). The concentration of total protein was quantified using a BCA assay, and total proteins were then separated by SDS‐PAGE. Subsequently, the blots were transferred onto polyvinylidene difluoride membranes (0.45 μm) (Thermo Fisher, Waltham, MA, USA), blocked with skim milk and incubated with the primary antibodies (Table 1) at 4°C overnight, while β‐actin (cellular and cytoplasmic controls) and histone H3 (nuclear control) served as loading controls. Immunoblots were washed thrice in TBS with Tween‐20, and the blots were incubated with corresponding secondary antibody at 37°C for 40 min. Then, the blots were visualized by using an ECL kit (Amersham Biosciences, Piscataway, NJ, USA).

**TABLE 1:** Primary antibodies used for Western blotting assay

Antibody	Dilution	Lot number	Manufacturer
Rabbit anti‐human STIP1 monoclonal antibody	1:1,000	A0036	ABclonal, Inc., Wuhan, China
Rabbit anti‐human NF‐κB p65 polyclonal antibody	1:1,000	AF5006	Affinity Biosciences; Changzhou, China
Rabbit anti‐human IκBβ polyclonal antibody	1:1,000	AF6448	Affinity Biosciences; Changzhou, China
Rabbit anti‐human TNF‐α polyclonal antibody	1:1,000	DF6080	Affinity Biosciences; Changzhou, China
Rabbit anti‐human IL‐6 polyclonal antibody	1:1,000	DF6087	Affinity Biosciences; Changzhou, China
Rabbit anti‐human histone‐H3 polyclonal antibody	1:1,000	17168‐1‐AP	Proteintech Group; Wuhan, China
Mouse anti‐human β‐actin monoclonal antibody	1:2,000	60008‐1‐I	Proteintech Group; Wuhan, China

### 2.5 HE staining

Spinal cord specimens were fixed with 4% paraformaldehyde, washed with flow water and dehydrated with ethanol and xylene. Then, the tissue was embedded into paraffin and cut into 5‐μm sections. Following deparaffination, the sections were stained with haematoxylin, differentiated with 1% hydrochloric acid/ethanol and counterstained with eosin. Finally, the sections were re‐dehydrated and mounted with gum. The pathological changes were observed with a microscope (Olympus, Tokyo, Japan) at a magnification of 200×.

### 2.6 Immunohistochemistry

Spinal cord specimens were made into paraffin‐embedded sections as described above. After deparaffination, the sections were reacted with antigen repair buffer at boiling for 10 min. The sections were blocked with goat serum and incubated with mouse anti‐human NeuN monoclonal antibody (1:100; lot number: ab104224; Abcam, Cambridge, UK), mouse anti‐human Iba‐1 monoclonal antibody (1:50; lot number: sc‐32725; Santa Cruz Biotechnology, CA, USA) or rabbit anti‐human STIP1 monoclonal antibody (1:100; lot number: AF9204, Affinity) at 4°C in the darkness overnight. After being washed in PBS, sections were incubated with the fluorescein isothiocyanate or Cy3‐conjugated IgG secondary antibody (Beyotime) at room temperature for 90 min. Finally, sections were counterstained with 4',6‐diamidino‐2‐phenylindole (DAPI) and mounted with the anti‐fading reagent. The images were captured with a fluorescent microscope at a magnification of 400×.

BV2 cells were pre‐seeded on glass slices, fixed with 4% paraformaldehyde for 15 min, hyalinized with 0.1% TritonX‐100, blocked with goat serum and incubated with rabbit anti‐human NF‐κB p65 polyclonal antibody (1:200; Affinity), or mouse anti‐human Iba‐1 monoclonal antibody (1:50; lot number: sc‐32725, Santa Cruz Biotechnology) at 4°C in the dark overnight. The cells were incubated with the Cy3‐conjugated IgG secondary antibody (Beyotime) at temperature and counterstained with DAPI. Finally, the slices were mounted with anti‐fading reagent and observed with a fluorescent microscope at a magnification of 400×.

### 2.7 Enzyme‐linked immunosorbent assay

The tumour necrosis factor (TNF)‐α and IL‐6 levels were measured in rat spinal cord specimens with Rat ELISA kits for TNF‐α or IL‐6 (USCN, Wuhan, China) following the manufacturer's protocols, and the TNF‐α and IL‐6 levels were quantified in the BV2 cell culture supernatant with Mouse ELISA kits for TNF‐α or IL‐6 (USCN) following the manufacturer's instructions.

### 2.8 Immunoprecipitation

BV2 cells were lysed with RIPA lysis buffer supplemented with 1% PMSF. The affinity agarose beads were pre‐coated with mouse anti‐human STIP1 monoclonal antibody (lot number: sc‐393475; Santa Cruz Biotechnology) and moue anti‐human HSPA8 (lot number: sc‐7298; Santa Cruz Biotechnology) or IgG antibody. The cell lysates were incubated with affinity agarose beads at 4°C for 2 h. After centrifugation, the sediment was collected and degenerated at boiling, and the supernatant was detected by SDS‐PAGE as previously described.

### 2.9 Ethical consideration

This study was approved by the Ethics Review Committee of Wuxi 9th Affiliated Hospital of Soochow University (approval number: KT2021011). All animal experiments were performed according to the Guide for the Care and Use of Laboratory Animals (8th, NIH) and the State Regulations for Laboratory Animal Management in China.

### 2.10 Statistics

All measurement data were described as mean ± SD, and all statistical analyses were performed using the software GraphPad Prism version 8.0 (GraphPad Software, Inc., La Jolla, CA, USA). Differences of means between groups were tested for statistical significance with Student's *t* test, and comparisons among multiple groups were performed with one‐way ANOVA followed by Bonferroni post hoc test. A *p* < 0.05 was considered statistically significant.

## 3 RESULTS

### 3.1 STIP1 expression is increased firstly followed by a reduction in rats after SCII

**FIGURE 1:**
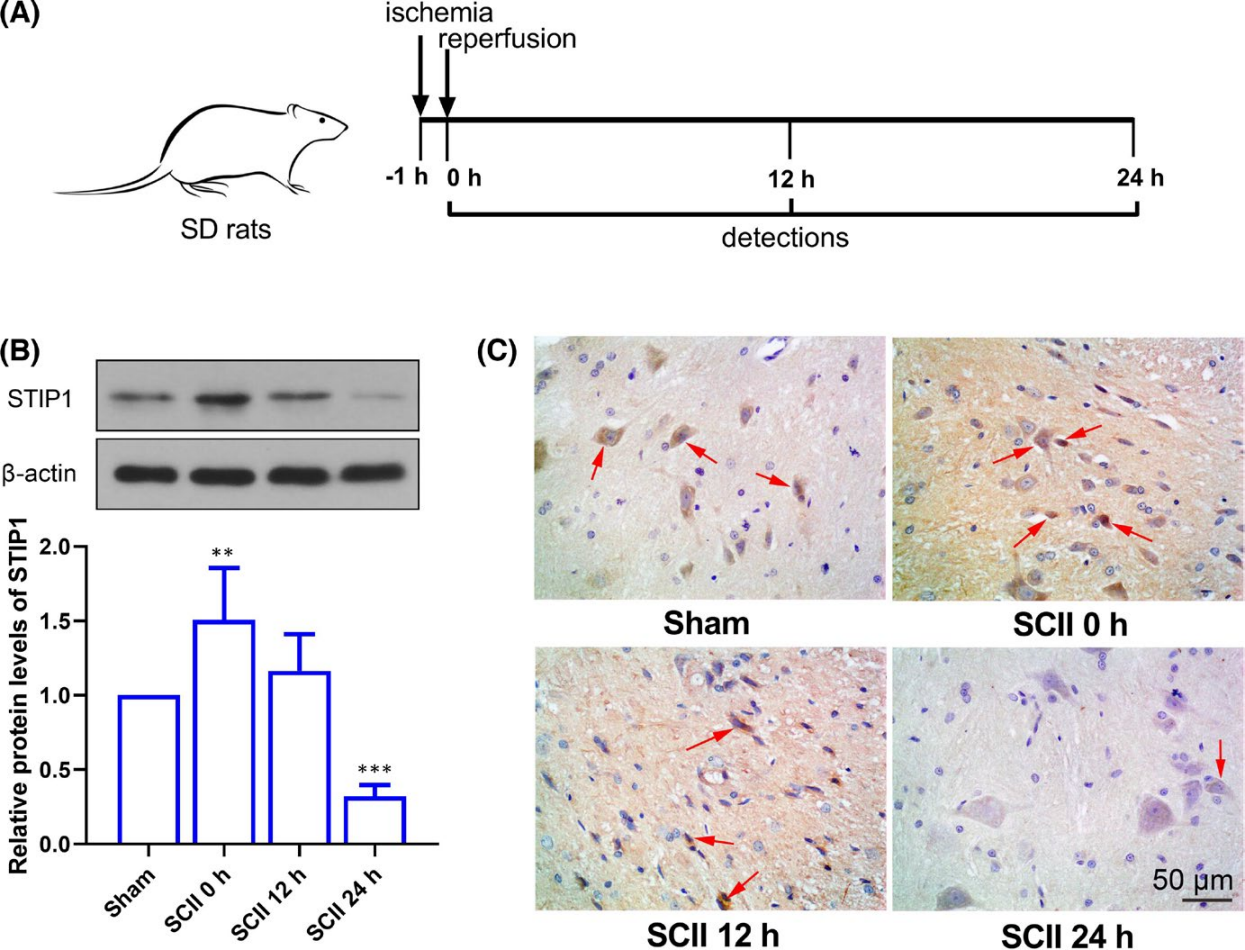
STIP1 expression increases firstly and then decreases in rats after SCII treatment. A, The rats undergo ischaemia by clamping abdominal aorta for 1 h and reperfusion for 24 h; B, Western blotting detects STIP1 expression in rat spinal cord specimens in absence or presence of SCII treatment; C, Immunohistochemical staining detects STIP1 expression in rat spinal cord specimens. The brown represents STIP1, and the STIP1‐postive cells are indicated by arrows. Scale bar =50 μm. SCII, spinal cord ischaemia‐reperfusion injury; STIP1, stress‐induced phosphoprotein 1. **p < 0.01, ***p < 0.001 versus the Sham group

We firstly determined STIP1 expression in rat spinal cord specimens after SCII treatment using Western blotting and immunohistochemistry (Figure 1A), and we found a rapid increase in STIP1 expression after ischaemia followed by a gradual reduction after reperfusion (Figure 1B,C). The STIP1 expression was significantly lower in the SCII group than in the Sham group 24 h post‐reperfusion (*p* < 0.001).

### 3.2 STIP1 alleviates ischaemia/reperfusion‐induced motor function impairment and neuronal injury in rat spinal cord

**FIGURE 2:**
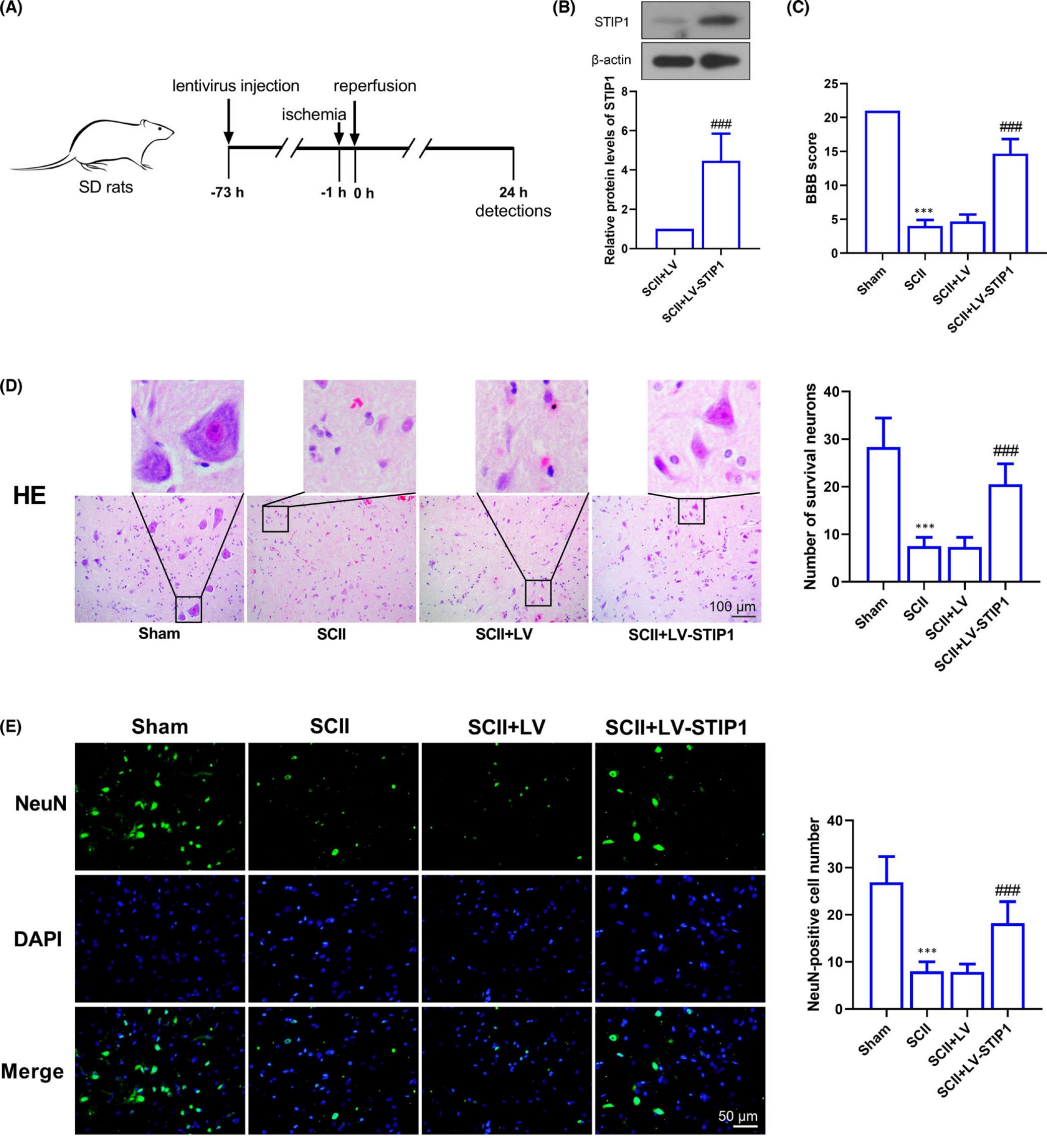
STIP1 alleviates ischaemia/reperfusion‐induced motor function impairment and neuronal injury in rat spinal cord. A, Ischaemia/reperfusion is given to rats; B, Western blotting detects STIP1 expression in rat spinal cord specimens after ST1P1 overexpression; C, BBB scoring measures the motor function of rat hind limbs; D, HE staining detects the morphological changes of rat spinal cord. Scale bar =100 μm; E, immunohistochemical staining detects the expression of the neuron marker NeuN. Scale bar =50 μm. BBB, Basso, Beattie and Bresnahan; ***p < 0.001 versus the Sham group, ^###^ *p* < 0.001 versus the SCII + LV group

To investigate the effect of STIP1 on SCII, the lentivirus containing STIP1 coding sequences was intrathecally injected into rats and rats underwent SCII treatment 72 h post‐injection (Figure 2A). Western blotting detected STIP1 overexpression in rat spinal cord specimens (Figure 2B), and SCII was found to cause conspicuous motor dysfunctions in rat hind limbs as revealed by BBB scoring (Figure 2C). HE staining showed loss of basophilic substances and decreased number of intact neurons in rat spinal cord specimens post‐treatment with SCII, and these morphological changes were recovered to some degrees after STIP1 overexpression (Figure 2D). Immunohistochemical staining showed that SCII caused loss of NeuN‐positive neurons, which was alleviated by STIP1 (Figure 2E).

### 3.3 STIP1 ameliorates ischaemia/reperfusion‐induced inflammation in rat spinal cord

**FIGURE 3:**
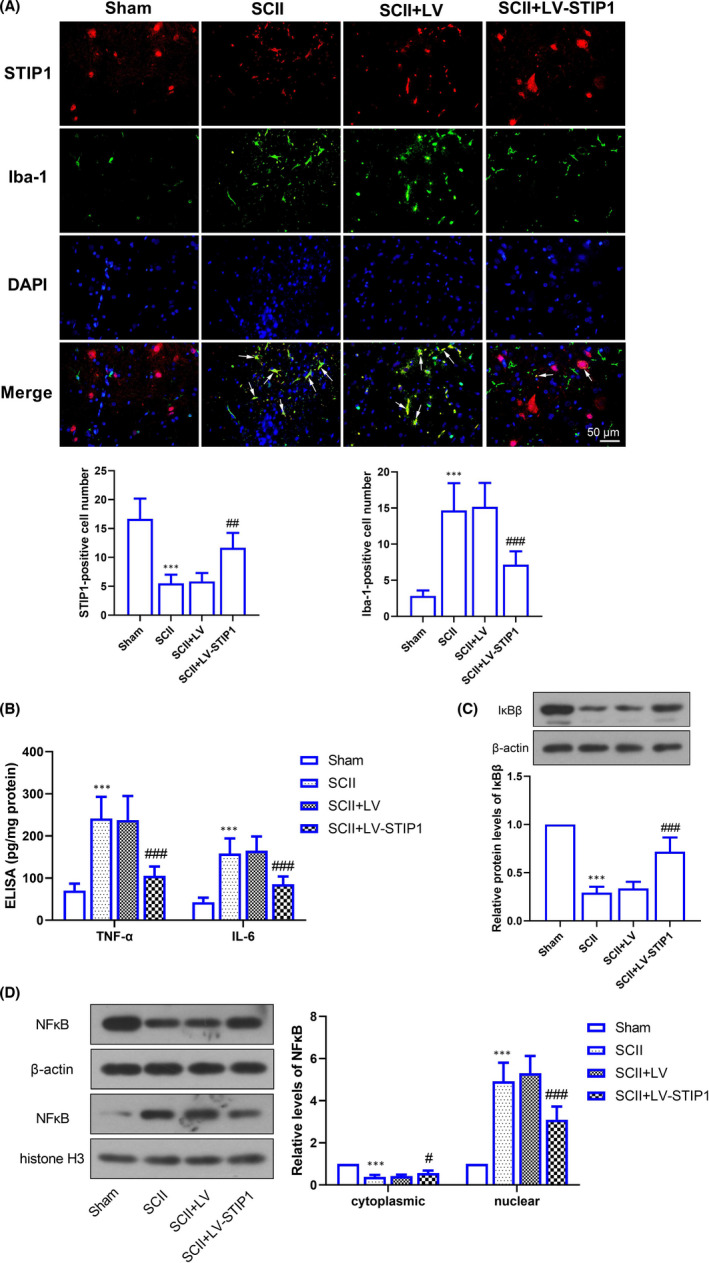
STIP1 ameliorates ischaemia/reperfusion‐induced inflammation in rat spinal cord. A, Immunohistochemical staining determines the expression and location of Iba‐1 and STIP1. The arrows indicate co‐location of STIP1 and Iba‐1. Scale bar =50 μm; B, ELISA detects TNF‐α and IL‐6 secretion in rat spinal cord specimens following SCII treatment and STIP1 overexpression; C, Western blotting determines IκBβ expression in rat spinal cord specimens; D, Western blotting determines the cytoplasmic and nuclear expression of NF‐κB p65. NF‐κB, nuclear factor kappa B; SCII, spinal cord ischaemia‐reperfusion injury; STIP1, stress‐induced phosphoprotein 1; TNF, tumour necrosis factor. ***p < 0.001 versus the Sham group; ^#^ *p* < 0.05, ^###^ *p* < 0.001 versus the SCII + LV group

Immunofluorescent staining detected elevated expression of Iba‐1, a marker of activated microglia, in rat spinal cord after ischaemia‐reperfusion treatment. Moreover, STIP1 was co‐located with Iba‐1, suggesting that STIP1 may be associated with microglia (Figure 3A). ELISA showed elevation of TNF‐α and IL‐6 levels in rat spinal cord following SCII treatment, and a decline after STIP1 overexpression (Figure 3B).

Then, we determined the expression of NF‐κB signalling‐associated proteins, and Western blotting revealed that SCII induced a reduction in IκBβ and cytoplasmic NF‐κB p65 expression and a rise in nuclear NF‐κB p65 protein in rat spinal cord, indicating activation of the NF‐κB signalling pathway (Figure 3C,D). Nevertheless, elevated IκBβ expression and reduced nuclear NF‐κB p65 expression were observed following administration with STIP1‐overexpressed lentivirus, suggesting that STIP1 deactivates NF‐κB signalling (Figure 3C,D).

### 3.4 STIP1 restrains OGD‐induced inflammation in microglial cells

**FIGURE 4:**
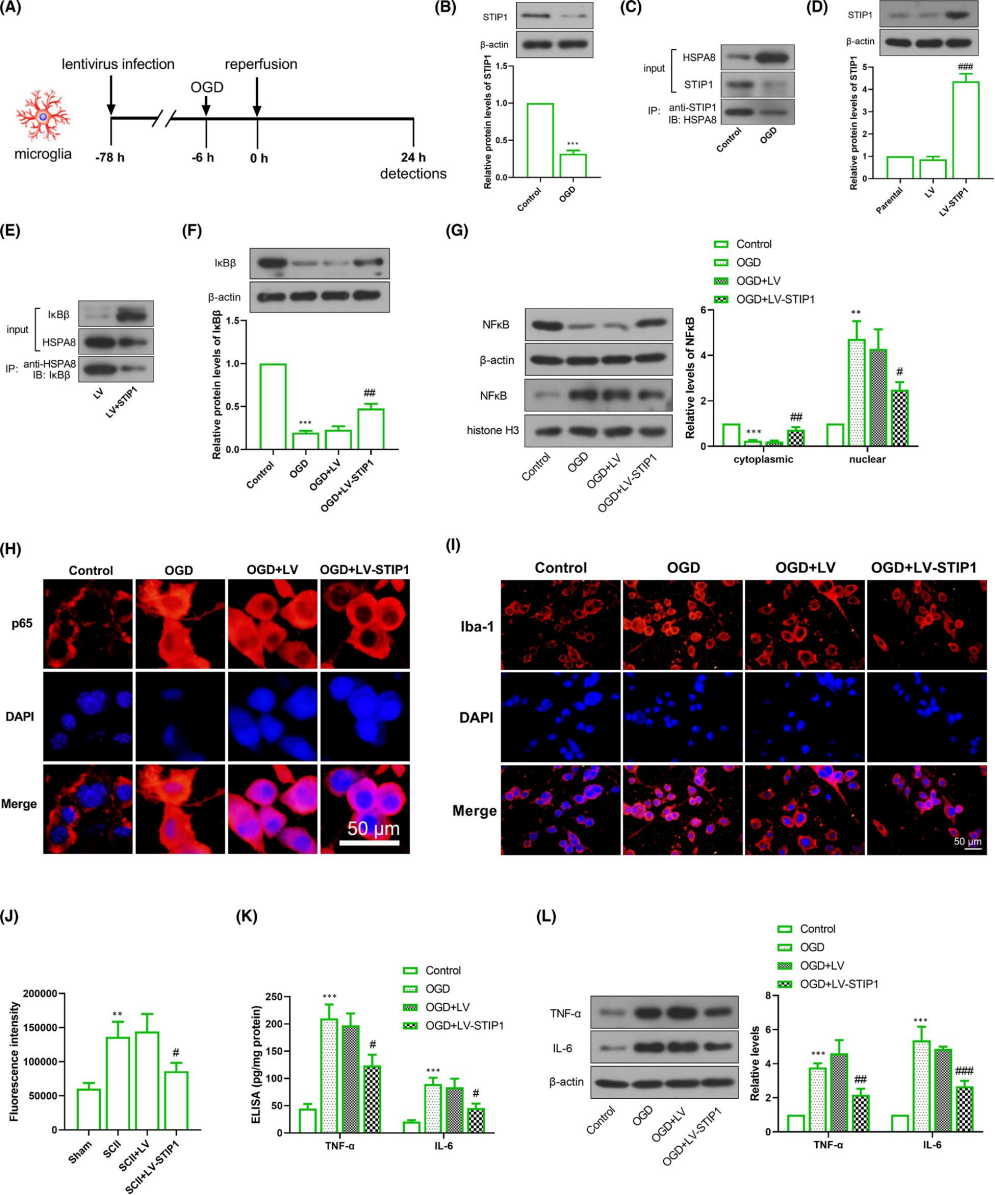
STIP1 restrains OGD‐induced inflammation in microglial cells. A, BV2 cell treatments; B, Western blotting determines STIP1 expression in BV2 cells after OGD treatment, C, Immunoprecipitation detects the binding between STIP1 and HSPA8 in BV2 cells; D, Western blotting determines STIP1 expression after STIP1 overexpression; E, Immunoprecipitation detects the interaction between HSPA8 and IκBβ in BV2 cells; F, Western blotting detects IκBβ expression in BV2 cells; G, Western blotting detects cytoplasmic and nuclear NF‐κB p65 levels in BV2 cells; H, Immunofluorescent staining detects the location of NF‐κB p65. Scale bar =50 μm; I, Immunofluorescent staining detects the expression of the microglia marker Iba‐1. Scale bar =50 μm; J, ELISA detects the secretion of inflammatory factors TNF‐α and IL‐6 in the cell culture supernatant; K, Western blotting determines TNF‐α and IL‐6 expression in BV2 cells after OGD treatment and STIP1 overexpression. NF‐κB, nuclear factor kappa B; OGD, oxygen and glucose deprivation; SCII, spinal cord ischaemia‐reperfusion injury; STIP1, stress‐induced phosphoprotein 1; TNF, tumour necrosis factor. **p < 0.01, ***p < 0.001 versus the Sham group, ^#^ *p* < 0.05, ^##^ *p* < 0.01, ^###^ *p* < 0.001 versus the SCII + LV group

Next, mouse microglia BV2 cells were used for detection of inflammation. First, BV2 cells were infected with the STIP1‐overexpressed lentivirus and underwent OGD for 6 h and reperfusion for 24 h (Figure 4A). Western blotting determined reduced STIP1 expression in BV2 cells following OGD treatment, which was similar to the findings seen in rats (Figure 4B). Immunoprecipitation demonstrated that OGD treatment caused a rise in HSPA8 expression, and STIP1 was found to bind to HSPA8 (Figure 4C), which was consistent with previous reports.^15^ Upon STIP1 overexpression, IκBβ expression was increased and HSPA8 expression was decreased. Moreover, the binding activity between IκBβ and HSPA8 was reduced (Figure 4D,E). Since HSPA8 was reported to destabilize IκBβ,^23^ it is therefore hypothesized that STIP1 enhance IκBβ expression through competitively binding to HSPA8 and attenuating its destabilizing effects on IκBβ. Western blotting and immunohistochemistry revealed that OGD treatment led to degradation of IκBβ and nuclear translocation of NF‐κB p65, which was abolished in BV2 cells to some degrees by STIP1 overexpression (Figure 4F–H). These results supported our hypothesis. Subsequently, we detected OGD‐induced inflammation, and immunohistochemical staining revealed that OGD‐induced Iba‐1 up‐regulation was recovered by STIP1 (Figure 4I). ELISA and Western blotting assays revealed that both the expression and secretion of TNF‐α and IL‐6 were promoted by OGD, and inhibited by STIP1 in BV2 cells (Figure 4J,K), which was consistent with the findings in rats.

## 4 DISCUSSION

To date, the role of STIP1 in SCII remains unclear. This study, designed in both in vivo and in vitro assays, aimed to examine the effect of STIP1 on SCII and unravel the possible mechanisms. In this study, we found a rapid increase in STIP1 expression followed by a decline in rat spinal cord following SCII treatment, and STIP1 overexpression alleviated SCII‐induced motor dysfunctions of hind limbs, and neuronal loss and inflammation in spinal cord of rats. In vitro assay showed activation of microglial cells after SCII treatment, and co‐location of STIP1 with microglia as revealed by immunoprecipitation, and STIP1 was found to restrain OGD‐induced inflammation in BV2 cells. In addition, our data showed that STIP1 activated NF‐κB signalling both in vivo and in vitro, and STIP1 led to elevated IκBβ expression, reduced HSPA8 expression and a reduced binding activity of IκBβ to HSPA8, suggesting that STIP1 competes with IκBβ to bind to HSPA8, and then suppresses NF‐κB signalling.

Nuclear factor kappa B signalling is a central signalling in immune responses. In mammals, the NF‐κB family is composed of five related transcription factors, including p50, p52, p65 (also termed RelA), c‐Rel and RelB. These transcription factors share an N‐terminal DNA‐binding/dimerization domain, known as the Rel homology domain responsible for DNA binding and homo‐ and heterodimerization. NF‐κB dimers bind to a variety of target DNA sequences called κB sites to modulate gene transcription through the recruitment of co‐activators and co‐repressors.^24^, ^25^ In most cells, NF‐κB complexes are inactive, residing predominantly in the cytoplasm in a complex with inhibitory IκB proteins (IκBα, IκBβ, IκBε, IκBζ, p100, p105, Bcl3 and IκBns). If the pathway is activated, the IκB protein is degraded and NF‐κB dimmers enter the nucleus to modulate target gene expression. The degradation of IκB is mediated by IκB kinase complex, which phosphorylates IκB and targets it for ubiquitin‐proteasome degradation.^24^ It has been recently reported that knockdown of HSPA8 inhibited degradation of IκB and deactivated NF‐κB signalling.^23^ In our study, STIP1 overexpression was found to cause an increase in IκB expression and deactivation of NF‐κB signalling. The binding between STIP1 and HSPA8 has been previously reported.^15^ We hypothesized that STIP1 competed with IκB to bind to HSPA8. STIP1 overexpression induced a decrease in HSPA8 binding to IκB, resulting in stabilization of IκB and deactivation of NF‐κB signalling. However, more studies are required to test our hypothesis.

Ischaemia‐reperfusion may elicit injury in many organs, such as myocardial infarction, ischaemic stroke, acute kidney injury, trauma, circulatory arrest, sickle cell disease and sleep apnoea.^26^ Ischaemia‐reperfusion is characterized by an initial restriction of blood supply followed by subsequent restoration of perfusion and concomitant reoxygenation.^26^ The restoration of blood flow and reoxygenation is frequently associated with an exacerbation of tissue injury and a profound inflammatory response.^27^ Ischaemia‐reperfusion activates various programmes of cell death, including necrosis, apoptosis and autophagy‐associated cell death.^26^, ^28^ NF‐κB is a redox‐sensitive transcription factor,^29^ and NF‐κB signalling is generally activated after ischaemia‐reperfusion.^30^ Inflammation and cell death may be modulated by NF‐κB signalling. In our study, ischaemia‐reperfusion induced loss of neurons and activation of NF‐κB signalling in rat spinal cord of rats, as well as activation of microglia. Microglia, a type of resident immune and macrophage cells,^31^ promotes neuro‐regeneration by regulating growth factors, and promotes phagocytosis and scavenges damaged spinal tissues.^32^ Microglia‐mediated inflammation contributes to scavenge of damaged tissues. However, excessive inflammatory reaction may aggravate the injury. Minocycline, a macrolide antibiotic that could inhibit microglial activation, was reported to preserve hind limb motor function and restrain inflammatory factor production in mice with SCII.^11^ Our data showed that STIP1 ameliorated ischaemia/reperfusion‐induced inflammation in rat spinal cord in vivo and OGD‐induced inflammation in microglial cells in vitro by inhibiting NF‐κB signalling activity, suggesting that STIP1 restrains SCII by inhibiting microglia‐mediated inflammation.

Stress‐induced phosphoprotein 1 may be produced by multiple cells, including astrocyte and microglia. In our study, STIP1 was found to be co‐located with Iba‐1, a marker of microglia, confirming that STIP1 was expressed in microglial cells of rat spinal cord. Because of limited microglia in spinal cord, the co‐localization of STIP1 and microglia was undetectable in rats in the Sham group. We detected elevated STIP1 expression followed by reduced expression in the rat spinal cord following ischaemia/reperfusion. However, a lot of neurons were lost, while microglia was activated in rats in the SCII group, leading to prominent co‐localization of STIP1 and microglia in SCII rats. Nevertheless, the changes of STIP1 expression were not clear in rat neurons. It is therefore considered that the difference between the number of neurons and microglial cells causes diverse STIP1 expression in neurons and microglial cells. STIP1 has been identified to play a neural protective role by binding to prion protein to inhibit the binding of soluble amyloid‐β oligomers to prion protein in mouse neurons.^12^ As a chaperone, STIP1 binds to HSPA8, which was also confirmed in our data. In addition, STIP1 induced an increase in IκB expression and a reduction in the binding of IκB to HSPA8, suggesting that STIP1 promotes IκB expression by occupying HSPA8 and then deactivates NF‐κB signalling. In addition, STIP1 expression rapidly increased after ischaemia and then decreased gradually after reperfusion. It is therefore speculated that the elevation of STIP1 may be protective in ischaemic conditions, and this elevation is broken off by reoxygenation. Our data demonstrated that STIP plays a protective role after ischaemia‐reperfusion in rat spinal cord and microglia, which supported our speculation.

## 5 CONCLUSIONS

In summary, the results of the present study demonstrate that STIP1 restrains ischaemia/reperfusion‐induced neuronal injury and inflammation by deactivating NF‐κB signalling in rats and mouse microglia. Our findings may provide novel insights for the diagnosis and treatment of SCI.

## CONFLICT OF INTEREST

The authors declare no conflict of interests.

## AUTHOR CONTRIBUTION

**Hongdou Jin:** Formal analysis (equal); Investigation (equal); Writing‐original draft (equal). **Xin Ge:** Conceptualization (equal); Funding acquisition (equal); Writing‐review & editing (equal). **Zhirong Huan:** Formal analysis (equal); Investigation (equal). **Hao Yao:** Investigation (equal). **Ce Xu:** Investigation (equal). **Jimin Cai:** Investigation (equal).

## CONSENT FOR PUBLICATION

All authors approve to publish this manuscript.

## Supporting information

File S1

## Data Availability

All data presented in this study are available upon request by contact with the corresponding author.
